# Hepatitis B Surface Antigen Loss and Hepatocellular Carcinoma Development in Patients With Dual Hepatitis B and C Infection

**DOI:** 10.1097/MD.0000000000002995

**Published:** 2016-03-11

**Authors:** Wan-Ting Yang, Li-Wei Wu, Tai-Chung Tseng, Chi-Ling Chen, Hung-Chih Yang, Tung-Hung Su, Chia-Chi Wang, Stephanie Fang-Tzu Kuo, Chen-Hua Liu, Pei-Jer Chen, Ding-Shinn Chen, Chun-Jen Liu, Jia-Horng Kao

**Affiliations:** From the Division of Gastroenterology (T-CT, C-CW), Department of Internal Medicine, Taipei Tzuchi Hospital, The Buddhist Tzuchi Medical Foundation; Division of Gastroenterology (H-CY, T-HS, C-HL, P-JC, C-JL, J-HK), Department of Internal Medicine; Graduate Institute of Clinical Medicine (C-LC, T-HS, C-HL, C-JL, J-HK); Hepatitis Research Center (W-TY, C-HL, J-HK); Department of Medical Research (J-HK); Department of Microbiology (H-CY), National Taiwan University College of Medicine and National Taiwan University Hospital; Master of Public Health Degree Program (W-TY), National Taiwan University, Taipei; Division of Gastroenterology (L-WW), Department of Internal Medicine, Taipei Medical University-Shuang Ho Hospital, New Taipei; School of Medicine (T-CT, C-CW), Tzu Chi University, Hualien; Genomics Research Center Academia Sinica (D-SC), Taipei, Taiwan; St Vincent's Hospital (SF-TK), Melbourne VIC, Australia; and Taiwan Liver Disease Consortium (TLC) (C-JL), Taipei, Taiwan.

## Abstract

Hepatitis B virus (HBV) and hepatitis C virus (HCV) infections are 2 major causes of chronic viral hepatitis. It is still unclear how HCV coinfection affects HBV replication and clinical outcomes in HBV/HCV coinfected patients.

We conducted a longitudinal study, which enrolled 111 patients with HBV/HCV coinfection and 111 propensity score-matched controls with HBV monoinfection. Both groups had comparable baseline age, sex, fibrosis stage, levels of HBV DNA, and HBV surface antigen (HBsAg). The HCV coinfection and other host/viral factors were correlated with various outcomes, including HBsAg loss and cirrhosis/hepatocellular carcinoma (HCC) development.

After a 10-year follow-up, we found that HCV coinfection itself was not associated with HBsAg loss. However, coinfected patients with alanine aminotransferase (ALT) level >80 U/L had a higher chance of HBsAg loss than those with ALT level ≤80 U/L [hazard ratio (95% confidence interval): 4.41 (1.75–11.15)] or matched controls with HBV monoinfection [hazard ratio (95% confidence interval): 3.40 (1.54–7.50)]. Besides, both HCV coinfection and higher ALT levels were associated with higher HCC risks and the HCC risks remained even after HBsAg loss in HBV/HCV con-infected patient.

HCV coinfection is not associated with HBsAg loss. A higher ALT level is a major determinant of HBsAg loss in patients with HBV/HCV coinfection. Both HCV coinfection and a higher ALT level were independent risk factors of HCC.

## Abstract 2

Supplemental Digital Content is available in the text

## INTRODUCTION

Chronic hepatitis B virus (HBV) and hepatitis C virus (HCV) infection are 2 major global health problems, which are associated with development of cirrhosis and hepatocellular carcinoma (HCC).^1^ In areas endemic for HBV and HCV infection, a substantial number of patients are infected with both viruses.^1^ Although several studies suggested HBV/HCV coinfected patients usually had a worse prognosis,^2,3^ it is still unclear whether HCV inhibits HBV replication, and how the interaction influences the clinical outcomes of the HBV carriers.

In patients with HBV monoinfection, HCC development is regarded as an end-stage liver disease while hepatitis B surface antigen (HBsAg) loss is considered functional cure.^4^ Previous longitudinal studies explored the effects of HCV coinfection by directly comparing the prognoses between patients with dual infection and those with HBV monoinfection.^2,3^ However, other cross-sectional studies have already shown that serum HBV DNA and HBsAg levels are usually lower in dually infected patients.^5–7^ Since HBV DNA and HBsAg levels are 2 major determinants of disease outcomes,^8–10^ it remains unclear whether differing clinical outcomes in dually infected patients are affected by HCV coinfection or by different baseline characteristics. Furthermore, it is unknown how the HCV, usually the latecomer, affects the replication of HBV, which is acquired perinatally, and how the interaction modifies the patients’ long-term outcomes. To this end, a well-designed case-control study to ascertain comparable HBV DNA and HBsAg levels between coinfected and monoinfected patients is warranted.

In this study, we conducted a retrospective cohort study, which enrolled HBV/HCV coinfected noncirrhotic patients as the case cohort, and HBV monoinfected patients, matched according to propensity score (PS), as the control cohort. The levels of all viral markers were first analyzed to identify factors associated with various clinical outcomes in the coinfected patients. The effect of HCV coinfection on clinical outcomes was further examined by comparing the case and control patients.

## METHODS

### Patient Enrolment

**FIGURE 1:**
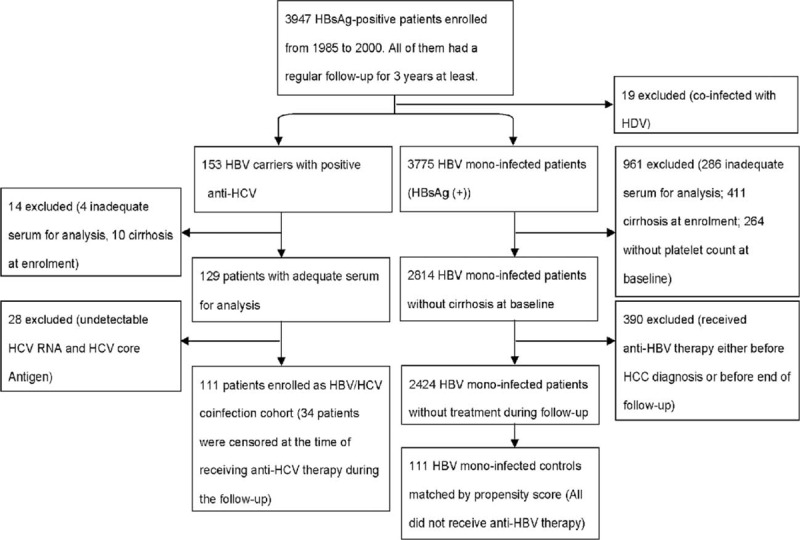
Flow of study participants.

Figure 1 shows how the patients were enrolled. All of them were aged >28 years with regular follow-up at the National Taiwan University Hospital. These patients exhibited no evidence of HDV and HIV coinfection and informed consents were obtained from all of them.

We enrolled 153 patients, who were positive for both HBsAg and antibodies against HCV (anti-HCV), consecutively from 1985 to 2000. After excluding patients without adequate serum samples for analysis (N = 4), patients with liver cirrhosis at the baseline (N = 10), and those without evidence of active HCV infection (N = 28), 111 patients with HBV/HCV coinfection were recruited. Among them, 34 patients were censored by initiating interferon (IFN) or pegylated IFN-based therapy. None of them had received nucleos(t)ide analog treatment.

The control group initially comprised 3775 patients with HBV monoinfection from the Elucidation of Risk Factors for Disease Control or Advancement in Taiwanese Hepatitis B cohort.^9^ We excluded patients who did not have adequate serum (N = 286) or platelet count at baseline (N = 264), patients who had cirrhosis at baseline (N = 411), and patient who received antiviral therapy during the follow-up (N = 390). Among the 2424 noncirrhotic HBV carriers who were free from treatment, 111 PS-matched control patients were selected for analysis.

### Data Collection

All patients were tested for serological markers (HBsAg, HBeAg, anti-HBe, anti-HCV, and anti-HDV), liver biochemical tests, and α-fetoprotein (AFP) at baseline. Throughout the follow-up period, serum alanine aminotransferase (ALT), AFP, and abdominal ultrasonography for HCC surveillance were examined every 3 to 6 months. The upper limit of normal (ULN) for serum ALT is 40 U/L. Serum samples collected at each visit were stored at −20°C until analysis.

### Definition and Diagnosis of HBsAg Loss, Cirrhosis, and HCC

HBsAg loss was defined as 2 consecutive HBsAg levels <0.05 IU/mL at least 1 year apart,^8^ as measured by the Architect HBsAg QT (Abbott Laboratories, Abbott Park, IL). Cirrhosis was diagnosed using histological or ultrasonographic findings in combination with clinical features.^11–13^ HCC was diagnosed using histological/cytological or radiological findings in hepatic nodules >1 cm.^14^

### Determination of Levels of HBV DNA and HBsAg, and HBV Genotype

HBV DNA was quantified using the Abbott RealTime HBV assay, 0.2-mL protocol (Abbott Laboratories, Abbott Park, IL), with a low detection limit of 15 IU/mL. HBV genotype was determined using a real-time polymerase chain reaction (PCR)-based single-tube assay as described previously.^15^ This method contains 2 consecutive steps. The first step uses a PCR to amplify the region (nt 1261–1600), and the second step uses a melting curve analysis to genotype HBV. The detection limit of this assay is approximately 200 IU/mL HBV DNA. An enzyme-linked immunosorbent assay (ELISA) kit, which detects genotype-specific epitopes in the pre-S2 region, was used to determine HBV genotype in patients with low viral loads.^16,17^ The HBsAg detection limit of the ELISA kit is approximately 100 IU/mL.

HBsAg levels were quantified using the Architect HBsAg QT assay with a low detection limit of 0.05 IU/mL.^18^ Serum samples collected at enrolment and the final follow-up were assayed initially. If the HBsAg levels were <0.05 IU/mL at the final follow-up, serum samples collected annually within the entire follow-up were then assayed to determine the time point of HBsAg loss.^8,19^

### Quantification of Serum HCV RNA, HCV Core Antigen, and HCV Genotyping

HCV RNA levels and HCV genotype were determined using a PCR-based assay complementary with melting curve analysis.^20^ The detection limit is 37 IU/mL (∼100 copies/mL). Since serum HCV RNA degrades during long-term storage,^21^ Architect HCV Ag assay (Abbott Laboratories, Abbott Park, IL) was used to determine HCV core Ag levels in patients who were positive for anti-HCV but had undetectable serum HCV RNA.^22^ The detection limit of the HCV Ag assay is equal to 15 IU/mL of HCV RNA and the HCV Ag levels can be linearly converted into HCV RNA levels because of high correlation between the 2 values (*r* = 0.9464).^22^

### Assessment of Fibrosis Stage

Liver fibrosis stage is known as an important factor affecting the development of HCC in patients with chronic viral hepatitis,^23–25^ thus this factor was included in the matching criteria. In this study, biochemical indexes were adopted to assess the fibrosis stage because liver biopsy, the gold standard for staging liver fibrosis, is not a routine procedure in clinical practice and fibroscan was not available when these patients were enrolled. Aspartate aminotransferase-to-platelet ratio index (APRI) and fibrosis index based on the 4 factors (FIB-4) are widely used as noninvasive tools to assess severity of liver fibrosis and a recent meta-analysis showed that FIB-4 is superior to APRI in HBV carriers.^26^ The FIB-4 index was calculated according to the formula: age (years) × AST [U / L]/(platelet counts [10^9^ / L] × (ALT [U/L])^1/2^), in which the age of the patient is the age at enrolment.^27,28^ Since our cohort included noncirrhotic patients only, the appropriate FIB-4 cutoff should differentiate F0/1 from F2/3. FIB-4 of 1.45 was chosen as a matching criterion since it has been used to define patients with significant fibrosis (separating F0/1 from F2, F3, and F4).^26^

### Statistical Analysis

Clinical follow-up commenced at the time of the first diagnosis. Person-years were censored on the date of death, the date of initiation IFN or pegylated IFN-based therapy (for coinfected patients), the final day of follow-up, or December 31, 2013. The cumulative incidence categorized by different variables was derived using Kaplan–Meier curve analysis, and a log-rank test was used to analyze statistical differences.

A PS matching method was used to minimize differences in baseline characteristics between the HBV/HCV coinfected group and the HBV monoinfected group.^29^ The matching members included 111 coinfected patients and 2424 HBV monoinfected patients. A multiple logistic regression analysis was used to calculate PS, the predicted probability of HCV coinfection, in each patient. The analysis included 5 variables: age, sex, HBV DNA levels, HBsAg levels, and FIB-4. After obtaining the PS for each patient, the greedy algorithm was used to match the cases to the control patients. The algorithm hierarchically sequences the matches so that the closest match appears first, followed by subsequent closest matches until no more matches can be made. Ideally, the closest matches would be patients matched to control patients to 5 digits of PS. The algorithm proceeds sequentially to a 1-digit match in PS. All PS-associated analyses were performed using SAS version 9.3 (SAS Institute Inc, Cary, NC).

Means and standard deviations (SD) were calculated for continuous variables, and percentages were calculated for categorical variables. HBV DNA (IU/mL), HBsAg (IU/mL), and HCV RNA (IU/mL) levels were logarithmically transformed for Pearson's correlation analysis. In patients with undetectable serum HBV DNA level, the lower limit of detection (15 IU/mL) was assigned for analysis.

A Cox proportional hazards model was used to calculate the crude and multivariable-adjusted hazard ratios (HR) and 95% confidence interval (CI) associated with HBsAg loss, cirrhosis, and HCC. All tests were 2-sided, with a significance level of 0.05. All analyses were performed using Stata statistical software (Version 11.2, Stata Corp, College Station, TX).

## RESULTS

### Baseline Characteristics

Table 1 lists the patients’ characteristics before and after PS matching. Marked differences existed between the 2 cohorts before matching. The HBV/HCV coinfected patients, compared to HBV monoinfected patients, were older (47.9 ± 12.3 vs. 41.4 ± 9.9 years), having a higher proportion of HBV DNA <2000 IU/mL (81.1% vs. 39.9%) and a higher proportion of HBsAg level <1000 IU/mL (67.5% vs. 40.2%). We selected 111 HBV monoinfected controls using PS for a fair comparison (Table 1). To be noted, approximately one-third of the coinfected patients had ALT levels >80 U/L, while ALT levels are usually within normal limits in the PS-matched monoinfected patients because most of them had HBV-DNA level <2000 IU/mL. We thus decided not to include ALT level as a matching criterion.

**TABLE 1:**
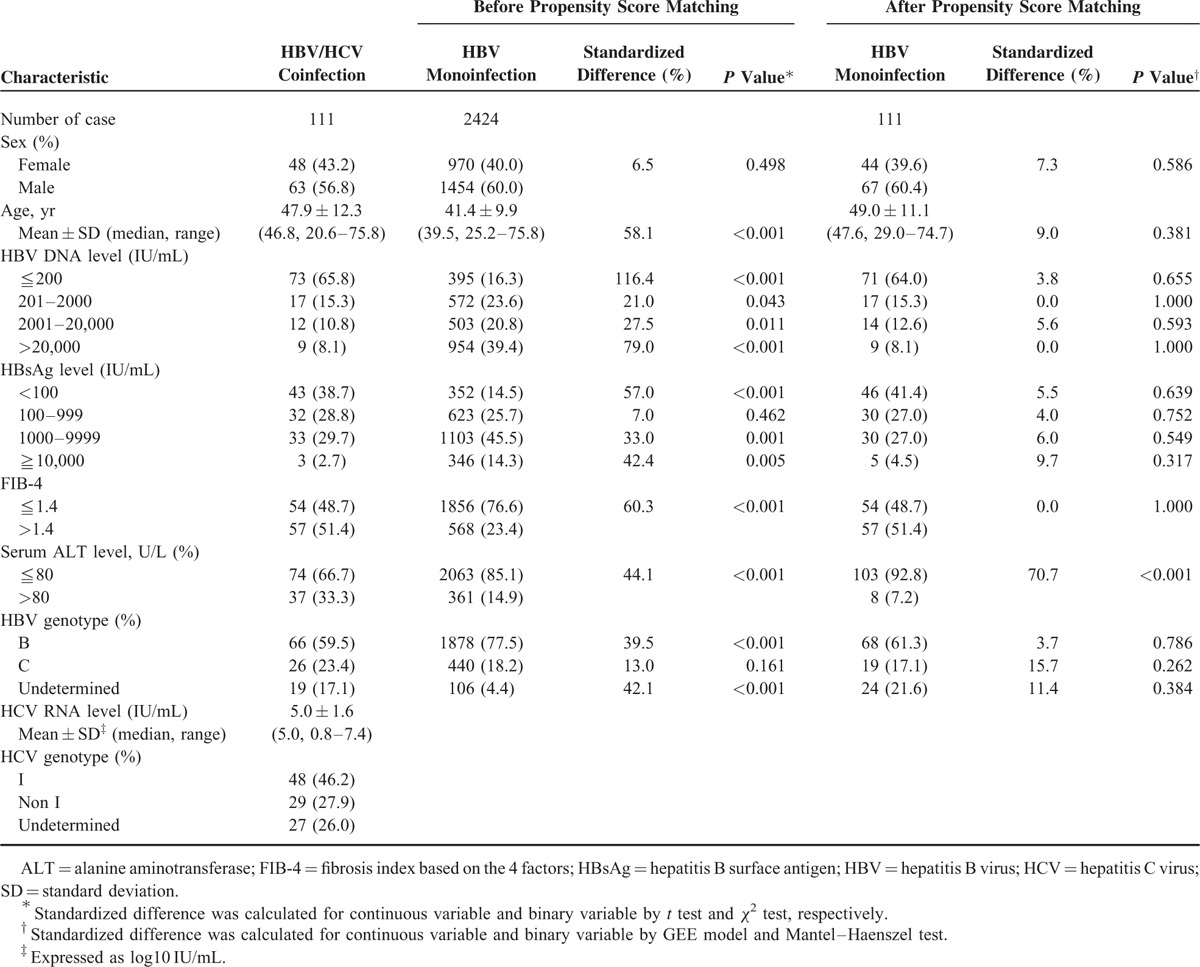
Baseline Characteristics of Case Patients With HBV/HCV Coinfection and Control Patients With HBV Monoinfection Before and After Propensity Score Matching

**FIGURE 2:**
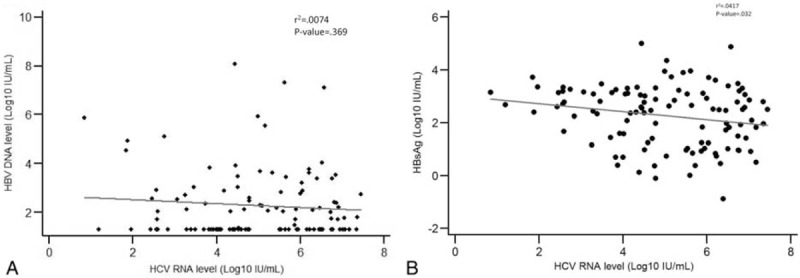
In 111 patients with HBV/HCV coinfection, (A) there is no correlation between levels of HCV RNA and HBV DNA, (B) but a weak inverse correlation between levels of HCV RNA and HBsAg.

For the coinfected cohort, the correlations between HCV RNA and HBV DNA/HBsAg levels were weak (Figure 2A and B).

### Follow-Up Data

Table 2 shows the follow-up data of each group. In the coinfection group, the annual incidence rates (95% CI) for HBsAg loss, HCC, and cirrhosis per 100 person-years were 1.70 (1.10–2.64), 1.51 (0.97–2.37), and 2.72 (1.92–3.87), respectively, with follow-up duration ranging from 10.3 to 11.3 years in average. Compared with the HBV monoinfection group, the coinfection group had higher cumulative incidence rates of HCC and cirrhosis.

**TABLE 2:**
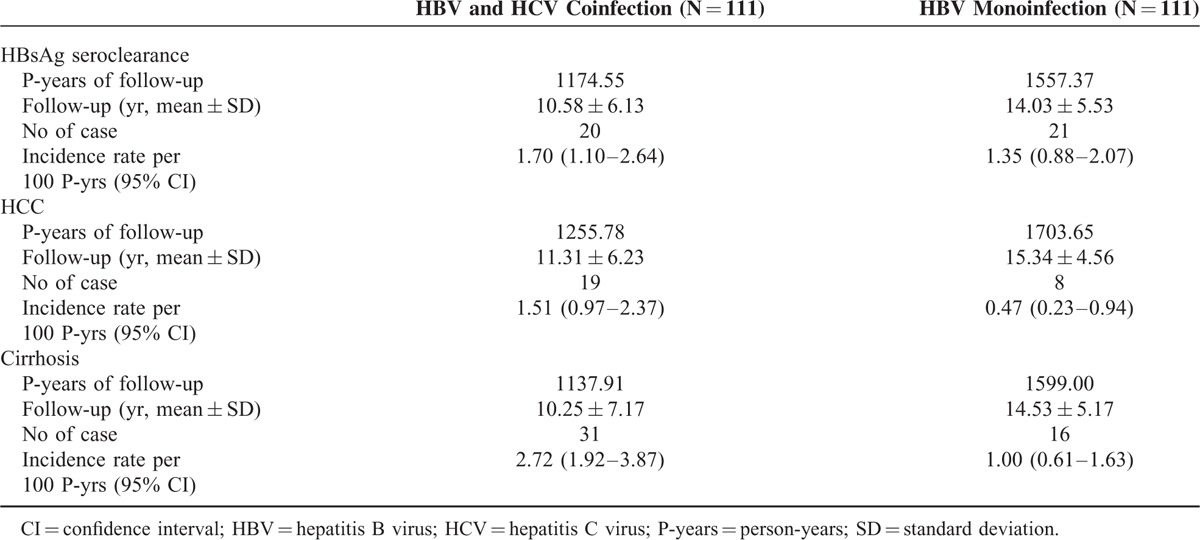
Cumulative Incidence of HBsAg Loss, HCC, and Cirrhosis in Cases With HBV/HCV Coinfection and Matched Controls With HBV Monoinfection

### Factors Affecting HBsAg Loss in Patients With HBV/HCV Coinfection

**FIGURE 3:**
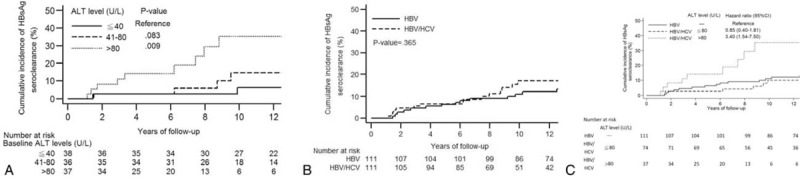
The impact of serum ALT level and HCV coinfection on spontaneous HBsAg loss. In the cohort of patients with HBV/HCV coinfection, (A) cumulative incidence of HBsAg loss can be stratified by serum ALT levels. In the cohort including coinfected patients and HBV monoinfected patients, cumulative incidence of HBsAg loss (B) is comparable between those with and without HCV coinfection, but (C) is higher in HCV coinfected patients with ALT level >80 U/L.

We first explored the factors affecting HBsAg loss in the coinfected patients. Lower serum HBV DNA and HBsAg levels were shown to be associated with increased incidence of HBsAg loss (Table 3). To evaluate the influence of baseline serum ALT levels, we categorized the patients by ALT levels into ≤40 U/L (1 × ULN), 41 to 80 U/L, and >80 U/L (2 × ULN) subgroups, which approximately divided the patients into tertiles (numbers of patients in the 3 categories were 38, 36, and 37, respectively, Figure 3A). In the subgroup of ALT >80 U/L, the mean (± SD) ALT level was 237.2 ± 214.5 U/L (median, 137 U/L; range, 82–960 U/L). This subgroup was associated with a higher cumulative incidence of HBsAg loss than the other 2 subgroups with lower ALT levels (Figure 3A).

**TABLE 3:**
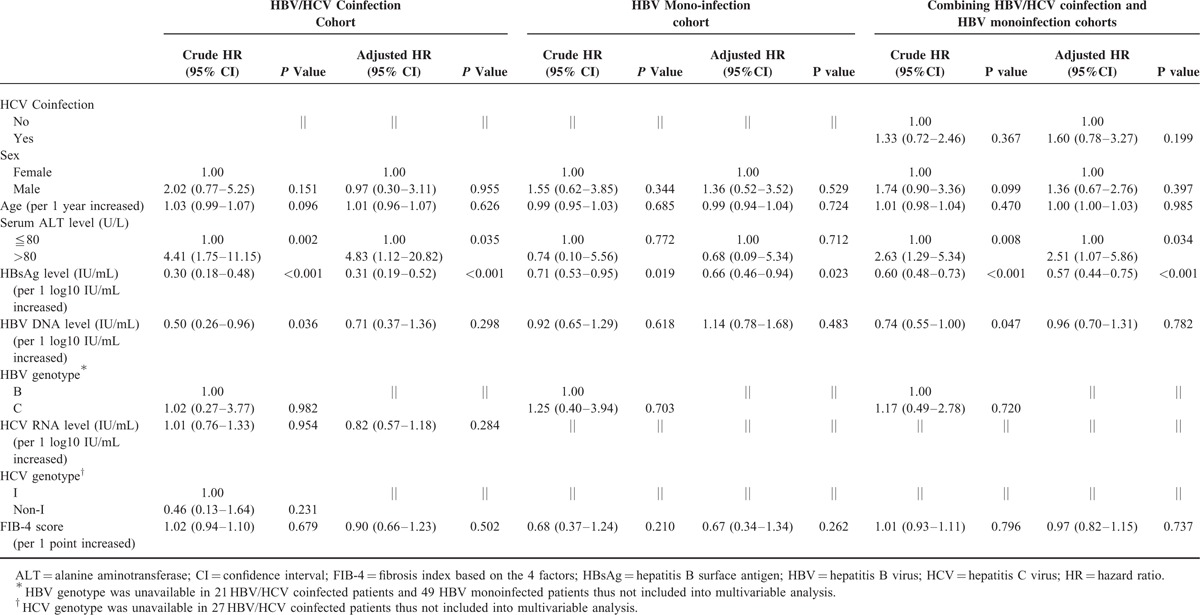
Univariable and Multivariable Analysis of Factors Associated With HBsAg Loss

The multivariable analysis showed that ALT level >80 U/L and lower HBsAg levels were associated with an increased incidence of HBsAg loss (Table 3). In contrast, HCV RNA level was not associated with HBsAg loss.

### Comparison of HBsAg Loss Between Coinfected Patients and PS-Matched HBV Monoinfected Patients

We compared the cumulative incidence of HBsAg loss between patients with coinfection and those with HBV monoinfection (Figure 3B). These 2 groups had a similar incidence rate of HBsAg loss. However, when we separated the coinfection patients into 2 subgroups by ALT level of 80 U/L, the subgroup with high ALT level was associated with a higher incidence of HBsAg loss than HBV monoinfection with HR of 3.40 (95% CI: 1.54–7.50) (Figure 3C). The multivariable analysis showed that ALT >80 U/L and a lower HBsAg level, but not HCV coinfection, were associated with increased incidence of HBsAg loss (Table 3). To be noted, only 8 HBV monoinfected patients had ALT level >80 U/L since 79.3% of HBV monoinfected patients had HBV DNA level <2000 IU/mL (Table 1). We thus could not define a subgroup of HBV monoinfection with high ALT level.

### Comparison of HBsAg Loss Between Both Groups of Patients With Low Viral Loads

To control HBV infection status, we conducted a subgroup analysis enrolling only patients who had serum HBV DNA levels <2000 IU/mL, including 88 patients with HBV monoinfection and 90 patients with HBV/HCV coinfection. We found that HCV coinfection was not associated with higher chance of HBsAg loss (HR: 1.43, 95% CI: 0.74–2.78) but ALT >80 U/L was associated with higher chance of HBsAg loss (HR: 2.66, 95% CI: 1.18–5.99).

### Factors Affecting HCC Development

**FIGURE 4:**
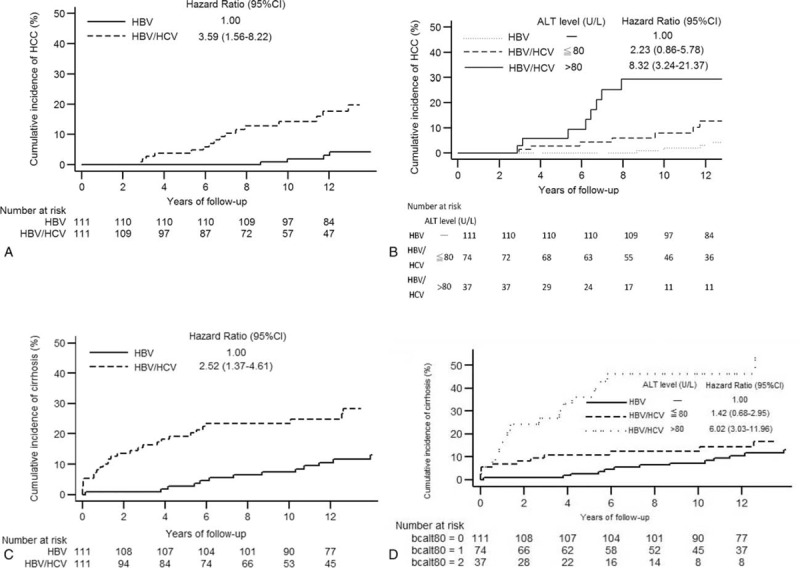
In the cohort including coinfected patients and HBV monoinfected patients, cumulative incidence of HCC is stratified (A) by HCV coinfection and (B) by the combination of serum ALT level and HCV coinfection; cumulative incidence of cirrhosis is also stratified (C) by HCV coinfection and (D) by the combination of serum ALT level and HCV coinfection.

The cumulative incidence rates of HCC development were then compared between the coinfection and HBV monoinfection groups. The patients with coinfection were associated with higher HCC risk than those with HBV monoinfection (Figure 4A). When further categorized by ALT level, the HCC risk was the highest in the coinfected patients with ALT level >80 U/L (Figure 4B). The multivariable analysis also showed that HCV coinfection, older age, and ALT level >80 U/L were 3 independent risk factors for HCC development (Supp Table 1).

### Factors Affecting Cirrhosis Development

Cirrhosis often precedes HCC development. We thus explored whether these 2 adverse events shared the similar risk factors. The coinfected patients were shown to be associated with higher cumulative incidence of cirrhosis than the monoinfected control patients (Figure 4C). In the coinfection group, patients with high ALT level and older age were associated with an increased risk of cirrhosis (Supp Table 2). When we categorized the overall patients by HCV coinfection status and ALT levels, the coinfected patients with ALT level >80 U/L were associated with the highest risk of cirrhosis (Figure 4D).

### Sensitivity Analysis: Prognostic Difference Between HBV/HCV Coinfected Patients and HBV Monoinfected Patients Matched for Serum ALT Levels

We included ALT level into our 5-variable PS matching model in the subgroup analysis to confirm the role of HCV coinfection. Seventy-six pairs of case and control were selected. We found that HCV coinfection was not associated with higher chance of HBsAg loss (HR: 1.06; 95% CI: 0.50–2.25), but increased the risks of HCC and cirrhosis development with HR of 3.85 (1.37–10.83) and 4.95 (1.82–13.47), respectively.

### Anti-HBs Development and ALT Levels in Patients With HBsAg Clearance

We analyzed anti-HBs development and ALT levels in patients who cleared HBsAg during the follow-up, including 20 HBV monoinfected patients and 21 HBV/HCV coinfected patients. The anti-HBs positive rates were comparable between monoinfected and coinfected patients (33.3% vs. 30.0%, *P* = 0.819). The ALT levels at HBsAg clearance were higher in coinfected patients than HBV monoinfected patients (mean ± SD, 59.5 ± 40.9 vs. 29.5 ± 22.9 U/L, *P* = 0.007).

### Prognosis After HBsAg Loss

HBsAg loss usually confers good prognosis in patients with HBV monoinfection, but it remains unclear how it affects the clinical outcomes in coinfected patients. The risks of cirrhosis and HCC (adverse events) were explored in coinfected patients after HBsAg being cleared. We excluded 4 coinfected patients who developed adverse events prior to HBsAg clearance and there were 16 coinfected patients and 21 HBV monoinfected patients cleared HBsAg. Subsequent to HBsAg loss, the coinfected patients experienced 3 adverse events (2 developed cirrhosis and 1 developed HCC) in 110.88 person-years (annual incidence, 2.71%), and the HBV monoinfected patients experienced 2 adverse events (both developed cirrhosis) in 142.09 person-years (annual incidence: 1.41%). The HR for an adverse event in HCV coinfection was 1.81 (95% CI: 0.30–10.92, *P* = 0.520).

## DISCUSSION

Previous cross-sectional and in vitro studies have suggested that HCV coinfection has an inhibitory effect on HBV replication,^1,30^ but the in vivo data do not support it.^31,32^ Our longitudinal cohort study, which adopted HBV monoinfected patients matched for viral factors (serum HBsAg and HBV DNA levels) and host factors (sex, age, and FIB-4 score) as controls, showed that HCV coinfection is not associated with HBsAg loss. The chance of HBsAg loss increased only in coinfected patients with higher ALT levels. These data suggested that liver injury induced by HCV, which is reflected by high ALT levels, might facilitate the immune response toward HBV clearance. On the other hand, the high ALT levels in coinfected patients also augmented the risks of HCC and cirrhosis. These findings lend support to the concept that the host immune response plays a role of the double-edge sword, which increases not only viral clearance but also liver damage.

There are two unique features of this study. The first unique part is the study design. It is known that transmission routes are very different between HBV and HCV in Taiwan.^1^ Patients with dual viral infection usually get HBV infection via perinatal transmission, while the HCV infection is mainly through blood transfusion, which occurs more frequently in aging population. It is evident that HBV/HCV coinfected patients were older when compared with HBV monoinfected patients, which is shown not only in our study cohort but also in REVEAL study, another prospective cohort study.^3^ In addition, we have already known from HBV's natural history that old HBV carriers are more likely to be inactive carriers, who are characteristic of low HBV DNA and HBsAg levels.^4^ To avoid the selection bias introduced by HCV transmission route, it is necessary to have comparable baseline virological characteristics between patients with dual- and monoinfection. We are the first time to conduct such a study since it is very rare to have a long-term follow-up of patients with dual viral infection and a large cohort with HBV monoinfection for control selection.

Second, our data is also the first time to show that HBsAg loss only increased in coinfected patients with high ALT level, which is mostly attributed to HCV superinfection. Most of coinfected patients had inactive HBV state, which is characterized by low HBV DNA levels.^5–7^ Unlike inactive HBV carriers, coinfected patients tend to have higher ALT levels, reflecting more liver necroinflammation caused by HCV infection. Although liver cell damage accelerates disease progression, it also facilitates HBV clearance. Such a phenomenon has also been observed in HBV carriers with coexisting steatohepatitis, which is another non-HBV cause of liver necroinflammation.^33^ In HBV carriers, our recent study has shown genotype C patients, compared with genotype B, have more hepatitis activity and a higher chance of HBsAg loss.^18^ Taking these lines of evidence together, we hypothesized that liver necroinflammation, which transforms the liver microenvironment more vulnerable to HBV-specific adaptive immune responses, could be the key to clear HBV infection.^34^ Furthermore, prolonged nucleos(t)ide treatment is the current treatment strategy to suppress the viral replication in HBV carriers. Our finding highlights a possible strategy to clear HBsAg by inducing limited necroinflammatory activity on top of nucleos(t)ide analogs. However, more studies are needed to prove this hypothesis.

It has been shown that HCV viral proteins, such as core protein, may induce hepatocarcinogenesis,^35,36^ which is supported by our finding that HCV coinfection increases HCC risk. However, whether HCV coinfection inhibits HBV replication remains dubious. Although the suppressive effect has been shown by overexpression of HCV core protein using in vitro study, other in vitro and in vivo studies have indicated that HCV coinfection does not inhibit HBV replication.^31,32^ Our study is the first longitudinal data to demonstrate that neither HCV RNA level nor HCV coinfection alone was associated with HBsAg loss, suggesting HCV itself may not directly interfere with HBV replication in humans.

Previous studies suggested that the risk of disease progression is negligible in noncirrhotic HBV carriers with HBsAg clearance.^37^ Our data shows that the risk of disease progression remained significant in the coinfected patients even after HBsAg clearance.

Our study had several limitations. First, the retrospective study design could be susceptible to selection bias. However, our previous finding derived from the HBV cohort has been validated well by REVEAL-HBV study, a prospective cohort study,^38^ which ensures our patient enrolment not biased. Second, 34 coinfected patients received IFN or pegylated IFN-based therapy during the follow-up, which might affect the long-term clinical outcomes.^39^ We censored these patients immediately after receiving antiviral treatment to minimize the possible biases. For HBV monoinfected cohort, we excluded 390 patients who received antiviral therapy.^4^ Since most of the included HBV control patients by PS matching were inactive carriers, the possible selection bias introduced by this exclusion is minimal. Third, ALT levels could not be matched in our original design and it is debatable whether the association is caused by HCV infection or by ALT levels. We performed a subgroup analysis, which included ALT as a matching criterion, and only 76 matches were enrolled. The data showed that HCV infection alone was not associated with HBsAg clearance, which supports our conclusion. Fourth, HCV RNA may degrade over time in storage.^21^ However, this limitation was unavoidable because a real-time method was not available until 2006. We used an HCV Ag test, which is less likely to degrade, in conjunction with a PCR assay in the current study to overcome this possible flaw.

In summary, HCV coinfection is not associated with HBsAg loss in patients with dual viral infection. However, the higher ALT levels is associated with a higher chance of HBsAg loss. Since viral replication can be suppressed by nucleos(t)ide analog treatment, our findings suggest that limited liver cell damage on top of the current treatment could be a new strategy for HBsAg seroclearance in HBV carriers.

## Supplementary Material

Supplemental Digital Content
