# Supplementary material for: Hepatitis B Surface Antigen Loss and Hepatocellular Carcinoma Development in Patients With Dual Hepatitis B and C Infection
Source: Medicine (Baltimore). 2016 Mar 11;95(10):e2995. doi: 10.1097/MD.0000000000002995 (PMC4998890; doi:10.1097/MD.0000000000002995)
Supplement: Supplemental Digital Content [file medi-95-e02995-s001.doc]

Supplementary Table 1. Univariable and multivariable analysis of factors associated with HCC by cox proportional hazards regression model

|  | HBV/HCV co-infected cohort | | | | Cohort including cases with HBV/HCV co-infection and matched controls with HBV mono-infection | | | |
| --- | --- | --- | --- | --- | --- | --- | --- | --- |
|  | Crude HR (95%CI) | P value | Adjusted HR (95%CI) | P value | Crude HR (95%CI) | P value | Adjusted HR (95%CI) | P value |
| HCV Co-infection  No  Yes | ║ | ║ | ║ | ║ | 1.00  3.59  (1.56-8.22) | .003 | 1.00  3.19  (1.26-8.05) | .014 |
| Sex  Female  Male | 1.00  1.06  (0.42-2.66) | .896 | 1.00  1.44  (0.52-4.00) | .485 | 1.00  1.07  (0.50-2.30) | .868 | 1.00  1.45  (0.62-3.35) | .389 |
| Age  (per 1 year increased) | 1.06  (1.02-1.10) | .002 | 1.08  (1.02-1.13) | .003 | 1.06  (1.03-1.10) | .001 | 1.08  (1.03-1.12) | <.001 |
| Serum ALT level (U/L)  ≦80  >80 | 1.00  3.43  (1.38-8.52) | .008 | 1.00  2.89  (1.01-8.21) | .047 | 1.00  4.68  (2.14-10.23) | <.001 | 1.00  2.71  (1.09-6.72) | .032 |
| HBsAg level (IU/mL)  (per 1 log10 IU/mL increased) | 1.11  (0.75-1.64) | .607 | 2.15  (1.14-4.08) | .019 | 1.20  (0.88-1.65) | .253 | 1.99  (1.24-3.21) | .004 |
| HBV DNA level (IU/mL)  (per 1 log10 IU/mL increased) | 0.90  (0.65-1.25) | .533 | 0.75  (0.50-1.13) | .175 | 0.94  (0.73-1.22) | .640 | 0.81  (0.60-1.09) | .162 |
| HBV genotype§  B  C | 1.00  3.03  (1.19-7.70) | .020 | ║ | ║ | 1.00  2.37  (1.08-5.17) | .031 | ║ | ║ |
| HCV RNA level (IU/mL)  (per 1 log10 IU/mL increased) | 0.96  (0.73-1.28) | .791 | 1.05  (0.77-1.44) | .746 | ║ | ║ | ║ | ║ |
| HCV genotype‡  I  Non-I | 1.00  1.90  (0.67-5.44) | .230 | ║ | ║ | ║ | ║ | ║ | ║ |
| FIB-4 score  (per 1 point increased) | 1.05  (0.99-1.11) | .136 | 1.00  (0.92-1.11) | .847 | 1.08  (1.02-1.14) | .006 | 1.02  (0.93-1.11) | .663 |

NOTE.

§ HBV genotype was unavailable in 21 HBV/HCV co-infected patients and 49 HBV monoinfected patients thus not included into multivariable analysis

‡ HCV genotype was unavailable in 27 HBV/HCV co-infected patients thus not included into multivariable analysis

Abbreviations: HBV, hepatitis B virus; HCV, hepatitis C virus; HR, hazard ratio; CI, confidence interval; ALT, alanine aminotransferase; HBsAg, hepatitis B surface antigen; FIB-4, fibrosis index based on the four factors.

Supplementary Table 2. Univariable and multivariable analysis of factors associated with cirrhosis by cox proportional hazards regression model

|  | HBV/HCV co-infected cohort | | | | Cohort including cases with HBV/HCV co-infection and matched controls with HBV mono-infection | | | |
| --- | --- | --- | --- | --- | --- | --- | --- | --- |
|  | Crude HR (95%CI) | P  value | Adjusted HR (95%CI) | P value | Crude HR (95%CI) | P value | Adjusted HR (95%CI) | P value |
| HCV Co-infection  No  Yes | ║ | ║ | ║ | ║ | 1.00  2.52  (1.37-4.61) | .003 | 1.00  2.16  (1.08-4.32) | .029 |
| Sex  Female  Male | 1.00  0.99  (0.49-2.04) | .988 | 1.00  0.96  (0.43-2.13) | .924 | 1.00  1.09  (0.61-1.95) | .775 | 1.00  1.20  (0.63-2.27) | .581 |
| Age  (per 1 year increased) | 1.07  (1.03-1.10) | <.001 | 1.04  (1.01-1.08) | .017 | 1.07  (1.04-1.09) | <.001 | 1.06  (1.03-1.09) | <.001 |
| Serum ALT level (U/L)  ≦80  >80 | 1.00  3.86  (1.87-7.97) | <.001 | 1.00  2.66  (1.13-6.26) | .025 | 1.00  3.58  (1.97-6.51) | <.001 | 1.00  1.82  (0.88-3.73) | .104 |
| HBsAg level (IU/mL)  (per 1 log10 IU/mL increased) | 0.79  (0.58-1.06) | .112 | 1.03  (0.69-1.51) | .901 | 0.93  (0.75-1.15) | .506 | 1.03  (0.78-1.37) | .824 |
| HBV DNA level (IU/mL)  (per 1 log10 IU/mL increased) | 0.88  (0.66-1.15) | .346 | 0.93  (0.68-1.29) | .667 | 1.00  (0.83-1.20) | .981 | 1.06  (0.84-1.33) | .625 |
| HBV genotype§  B  C | 1.00  2.22  (1.05-4.72) | .038 | ║ | ║ | 1.00  2.08  (1.11-3.88) | .022 | ║ | ║ |
| HCV RNA level (IU/mL)  (per 1 log10 IU/mL increased) | 1.03  (0.82-1.30) | .790 | 1.00  (0.77-1.29) | .999 | ║ | ║ | ║ | ║ |
| HCV genotype‡  I  Non-I | 1.00  1.27  (0.54-2.98) | .578 | ║ | ║ | ║ | ║ | ║ | ║ |
| FIB-4 score  (per 1 point increased) | 1.07  (1.03-1.12) | .001 | 1.05  (0.98-1.11) | .166 | 1.09  (1.05-1.13) | <.001 | 1.05  (1.00-1.11) | .057 |

NOTE:

§ HBV genotype was unavailable in 21 HBV/HCV co-infected patients and 49 HBV monoinfected patients thus not included into multivariable analysis

‡ HCV genotype was unavailable in 27 HBV/HCV co-infected patients thus not included into multivariable analysis

Abbreviations: HBV, hepatitis B virus; HCV, hepatitis C virus; HR, hazard ratio; CI, confidence interval; ALT, alanine aminotransferase; HBsAg, hepatitis B surface antigen; FIB-4, fibrosis index based on the four factors.
